# The Impact of Physical Activity and Nutritional Patterns on Phase Angle in Healthy Adolescents

**DOI:** 10.3390/nu18030516

**Published:** 2026-02-03

**Authors:** Agata Przytula, Paweł Glibowski, Joanna Popiolek-Kalisz

**Affiliations:** 1 Department of Clinical Dietetics, Medical University of Lublin, ul. Chodzki 7, 20-063 Lublin, Poland; agata.przytula@umlub.pl; 2 Department of Biotechnology, Microbiology and Human Nutrition, University of Life Sciences in Lublin, 8 Skromna Str., 20-704 Lublin, Poland

**Keywords:** physical activity, phase angle, malnutrition, children, body composition, bioelectrical impedance analysis

## Abstract

Background: Phase angle (PhA), derived from bioelectrical impedance analysis (BIA), is a non-invasive parameter that reflects cellular integrity and nutritional status. Although PhA is increasingly used in pediatric settings, evidence on modifiable determinants in healthy adolescents remains limited. Methods: This study was conducted in 56 adolescents (median age 16 years) who underwent BIA measurement including PhA at 50 kHz. Lifestyle and diet were assessed using the validated questionnaire and included physical activity level (school and free time), sleep length, and diet quality indices together with selected food intake frequencies. Results: The median PhA was 5.16° (IQR 4.88–5.46). In multivariable models, male sex (B = 0.96, *p* < 0.001) and higher free-time physical activity were independently associated with higher PhA, with graded effects for moderate (B = 0.42, *p* = 0.004) and high activity (B = 0.55, *p* = 0.001) versus low. Dietary indices and individual food items did not retain significance after adjustment. Penalized logistic models confirmed lower odds of low PhA with moderate (OR: 0.13, 95% CI: −3.66 to −0.56) and high (OR: 0.01, 95% CI: −9.15 to −1.87) versus low free-time activity. Conclusions: In healthy adolescents, habitual free-time physical activity is the main factor of PhA. These findings support the promotion of physical activity in youth as a modifiable determinant of cellular health.

## 1. Introduction

Adolescence is a critical period during which health behaviors consolidate and shape future adult health trajectories. Beyond conventional anthropometric parameters, phase angle (PhA) derived from bioelectrical impedance analysis (BIA) has emerged as a promising marker of nutritional and functional status at the cellular level, as it integrates resistance and reactance to reflect cell membrane integrity [1]. PhA has been shown to correlate with muscle strength and overall health-related fitness, and lower values are consistently linked to malnutrition and adverse clinical outcomes [2,3]. Recent research also suggests that PhA may distinguish between nonathletes and student athletes, reflecting differences in training status and muscle quality not explained by BMI or fat-free mass [4].

Globally, insufficient physical activity among adolescents has been recognized as a major public health concern, with the World Health Organization reporting that over 80% of teenagers do not meet recommended activity levels [5]. This inactivity, combined with Western dietary habits, contributes to early manifestations of metabolic risk [6]. Experimental research shows that even moderate amounts of activity can benefit high-risk youth (e.g., those with obesity) [7,8]. Children and adolescents (5–17 years) should accumulate an average of at least 60 min per day of at least moderate-intensity physical activity [9]. Some health benefits can be achieved with an average of 30 min per day. Moreover, higher-intensity activities, including those that strengthen muscle and bone, should be incorporated or added whenever possible. Aerobic activities should constitute the majority of physical activity. Muscle- and bone-strengthening activities should be included at least 3 days per week. This underscores that promoting sufficient volume, intensity, and variety of activity is crucial for improving the health, nutritional status, and body composition of adolescents [10]. Higher amounts and intensity of physical activity, as well as different types of activity (such as aerobic exercise and muscle- and bone-strengthening activities), are associated with improved health outcomes [9].

Moreover, a recent study in Central Europe examined the links between sleep and eating habits and indicators of somatic health in young adults and showed significant correlations between eating and sleeping patterns and indicators of somatic health in young men and women. In men, the more frequent consumption of salt and salt-rich foods led to lower PhA. Eating fewer meals during the day, eating breakfast more often, and increasing fruit and vegetable consumption were associated with higher PhA. The authors underlined the need for further studies in more diverse populations to confirm these associations and determine their causal nature and clinical significance [11]. Furthermore, a Polish study in university students showed a significant association between physical activity and PhA [12]. The school routine creates a distinctive environment for lifestyle behaviors. That is why, to address this gap in adolescents, our study evaluates both habitual free-time activity and detailed diet quality indices, thus offering a more nuanced approach to the determinants of phase angle in this age group. This issue is particularly important as available evidence indicated significant changes in dietary patterns and physical activity in children in the Central Europe region after the COVID-19 pandemic [13].

Previous studies have mainly examined PhA in clinical or pediatric populations with obesity, where associations with muscle strength and fitness were observed [7,14,15]. However, evidence in healthy, community-based adolescents remains scarce, and the combined role of diet quality and habitual free-time physical activity in shaping PhA has not yet been addressed. That is why we investigated if diet quality and habitual physical activity are associated with PhA in a cohort of healthy adolescents.

## 2. Materials and Methods

This cross-sectional observational study enrolled healthy volunteer students aged 14–19 years. Exclusion criteria were chronic illness, medication affecting body composition, or recognized contradictions to BIA measurement.

Body composition, including PhA at 50 kHz, was measured using a multifrequency analyzer (Tanita MC-780, Tanita Corporation, Tokyo, Japan) by trained staff following the manufacturer’s instructions. BIA involves the application of low-intensity alternating current at various frequencies, which flows through body fluids and tissues via electrodes placed on the skin. Because different tissues conduct electrical current with different efficiency, BIA enables the estimation of resistance and reactance. On that basis, the device algorithms then calculate fat mass and fat-free mass [1]. While the multifrequency mode is used to improve body composition calculations, PhA is a direct bioelectrical parameter, less prone to errors than calculated body composition parameters [16], and measurement at 50 kHz frequency is a recognized golden standard in nutritional status assessment [17]. This approach maximized the reliability and comparability of our results across the literature. Our research focused on overall cellular health and membrane integrity assessment, for which, as already mentioned, 50 kHz is a recognized marker [18]. PhA reflects cell membrane integrity and is regarded as a raw bioelectrical parameter of nutritional and functional status. Prior to the measurements, participants were asked to adhere to standard pre-test recommendations. These included abstaining from vigorous physical activity for at least 12 h; avoiding caloric food and beverages for 7–8 h [16,19]; refraining from caffeine and energy drinks on the test day; ensuring adequate hydration while avoiding excessive fluid intake immediately beforehand; and emptying their bladder within 30 min of the test. Following the device manufacturer’s guidelines, all measurements were conducted in the morning, with participants wearing light clothing and in a standing position. The BIA method has several contraindications: it should not be performed in pregnant women, in patients with implanted pacemakers, during high fever, in individuals with open wounds, or after amputations, since these conditions may interfere with current flow.

Diet and lifestyle were assessed with the validated questionnaire, which allows for the evaluation of food intake and lifestyle habits over the preceding 12 months. The questionnaire consists of four sections: eating habits, including questions on typical food choices; meal frequency; beliefs and opinions about food and nutrition; and lifestyle and sociodemographic data [20]. This structure enables a comprehensive assessment of habitual diet and related behaviors in adolescents. From the dietary part of the questionnaire, three indices were derived: the Pro-Healthy Diet Index (pHDI), the Non-Healthy Diet Index (nHDI), and the Diet Quality Index (DQI). The pHDI is based on the reported frequency of consumption of 10 food groups with a potentially beneficial impact on health, including whole-grain bread, buckwheat/oatmeal, milk, fermented dairy drinks, quark, white meat, fish, legumes, fruits, and vegetables. Scores range from 0 to 20 and are often standardized to a 0–100 scale. Conversely, the nHDI reflects the frequency of consumption of 14 food groups considered detrimental to health, such as white bread, refined pasta/rice, fast food, fried dishes, butter, lard, cheese, processed meat, red meat, sweets, canned meats, sugary soft drinks, energy drinks, and alcohol. Scores range from 0 to 28, also typically converted to a 0–100 scale. Together, the pHDI and nHDI provide a quantitative overview of both beneficial and adverse dietary patterns. DQI is a composite measure integrating both pHDI and nHDI. It includes 24 food groups and applies weighting coefficients to their consumption frequency, providing a score from −100 to +100. Interpretation follows three categories: −100 to −25 reflects a diet dominated by unhealthy choices; −24 to +24 indicates a diet with a low intensity of both healthy and unhealthy features (neutral); and +25 to +100 reflects a diet with predominantly healthy characteristics. This index provides a single, comprehensive measure of overall dietary quality, positioning each participant on a continuum from very unhealthy to very healthy [21].

Physical activity was evaluated with separate questions on activity during school hours and during free time. In Poland, the school day is predominantly organized into consecutive classroom-based lessons separated by short breaks, during which students typically move between classrooms and school areas according to the timetable. In the present study, physical activity at school was assessed as an overall construct using the self-reported school activity variable, intended to reflect the respondent’s general activity level during school time and in school-related settings. This construct encompasses structured and unstructured components, including scheduled physical education lessons, routine locomotion (e.g., walking between classrooms, stair use, movement during breaks), and, if taken, school-based extracurricular activities. In parallel, physical education class attendance was recorded separately (Yes/No) to characterize participation in timetabled physical education lessons. These measures were collected in parallel because physical education attendance does not necessarily translate into similar overall activity, as the volume and intensity of activity at school may differ substantially between students despite comparable exposure to compulsory physical education. For school-related activity, respondents classified their typical level as low (spending more than 70% of the time sitting), moderate (approximately equal proportions of sitting and movement), or high (spending about 70% of the time in motion or engaging in physically demanding work). Students answered the question of whether they exercise during physical education classes at school or whether they have been or are currently exempt from these classes. In total, 17.4% of students answered that they do not exercise during classes at school. For leisure-time activity, three categories were also distinguished: low (predominantly sedentary behaviors such as watching television, reading, light household chores, and occasional walking 1–2 h per week), moderate (regular light activities such as walking, cycling, gymnastics, or gardening for 2–3 h per week), and high (more intensive activities such as cycling, running, or structured sport, performed regularly).

Sleep duration was assessed separately for weekdays and weekends and categorized into three levels: short (<6 h), adequate (6–9 h), and long (>9 h). This allowed us to capture both habitual physical activity and sleep patterns as potential lifestyle determinants of phase angle.

Statistical analysis was performed using R statistical software (version 4.4.2, R Foundation for Statistical Computing, Vienna, Austria). Continuous variables were tested for normality using the Shapiro–Wilk test and are presented as the mean ± standard deviation (SD) or median with interquartile range (IQR), as appropriate. Categorical variables are expressed as counts and percentages. Associations between PhA and lifestyle or dietary factors were initially explored in univariate linear regression models. Variables with a *p*-value < 0.10 in univariate testing were subsequently entered into multivariable linear regression models to identify independent predictors of PhA. To further evaluate the associations and address potential bias in small samples, Firth’s penalized logistic regression was applied, with low PhA (below median) as the dependent variable. Model performance was assessed by adjusted R^2^ for linear regression and likelihood ratio tests for logistic regression. A two-sided *p*-value < 0.05 was considered statistically significant.

The study was approved by the local Bioethics Committee of Medical University of Lublin. The participants and their parents (legal guardians) signed a consent form for their participation in the study.

## 3. Results

**Figure 1:**
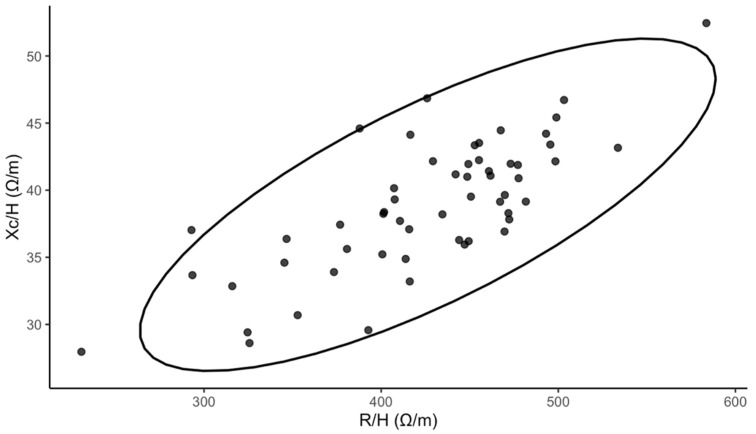
Bioelectrical impedance vector analysis (Xc—reactance, R—resistance, H—height, ellipse depicts the descriptive 95% dispersion region).

Fifty-six adolescents were enrolled in the study (median age 16 years; 85.7% female). The median BMI was 20.63 kg/m^2^ (18.81–22.68). The median PhA was 5.16° (4.88–5.46). For free-time physical activity, 30.4% reported low, 39.1% reported moderate, and 30.4% reported high levels. Details of baseline characteristics are presented in Table 1. To enhance transparency regarding impedance-derived measurements beyond summary PhA values, we additionally visualized the bioelectrical impedance vector analysis in Figure 1.

In univariate analyses, male sex and higher free-time physical activity were positively associated with PhA. Several individual food items showed positive associations in unadjusted models; BMI showed a weak positive association. Physical activity at school and sleep duration were not significant correlates. Diet indices (pHDI, nHDI, DQI) were not associated with PhA in unadjusted models. A complete summary of univariate regression results is presented in Table 2.

**Figure 2:**
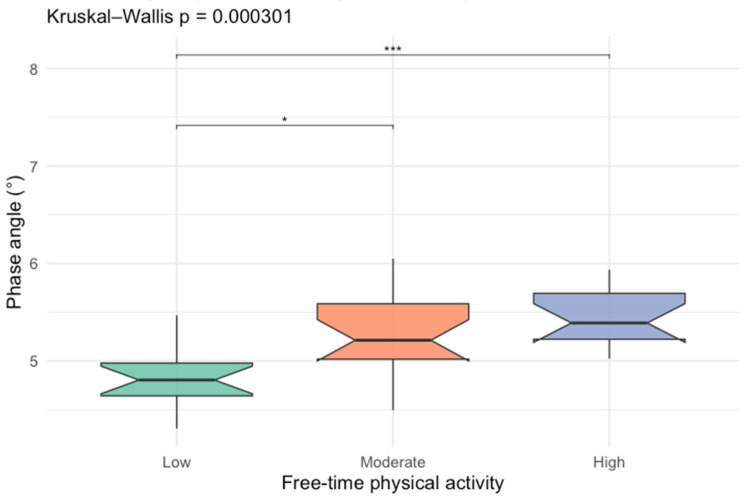
Box-plots comparing phase angle values between different free-time activity levels (* *p* < 0.05; *** *p* < 0.001).

After identifying leisure-time physical activity as an important factor of PhA, we compared PhA between different activity levels, which is presented in Figure 2.

To identify independent predictors of PhA, a series of multivariable linear regression models were constructed. The full model included sex, BMI, physical activity during free time, and selected food items that demonstrated significant associations in univariate analyses. The model was statistically significant overall (F(12, 33) = 5.48, *p* < 0.001) and explained a substantial proportion of variance in PhA (R^2^ = 0.666; adjusted R^2^ = 0.545), with residual standard error = 0.404. In this model, male sex was independently associated with higher PhA (B = 0.897, 95% CI 0.464 to 1.330; *p* < 0.001). In addition, high physical activity during free time was independently associated with higher PhA (B = 0.530, 95% CI 0.151 to 0.909; *p* = 0.0076), while moderate free-time activity showed a borderline association (B = 0.306, 95% CI −0.022 to 0.635; *p* = 0.0663). BMI was not independently associated with PhA after adjustment (B = 0.012, 95% CI −0.033 to 0.057; *p* = 0.6012), and none of the dietary variables retained significance in the multivariable model (all *p* > 0.05). Collectively, these findings indicate that, within the covariate set examined, sex and habitual leisure-time activity were the primary independent correlates of PhA, whereas the observed univariate dietary signals were not robust to adjustment. Details of the model are presented in Table 3.

A reduced model was then fitted including only sex and free-time physical activity, given that dietary variables did not retain significance in the fully adjusted model. The reduced model was statistically significant overall (F(3, 42) = 23.09, *p* < 0.001) and explained a substantial proportion of variance in PhA (R^2^ = 0.623; adjusted R^2^ = 0.596), with a residual standard error of 0.381. In this model, male sex remained strongly associated with higher PhA (B = 0.962, 95% CI 0.655 to 1.268; *p* < 0.001). Relative to low free-time activity, both moderate and high free-time physical activity were independently associated with higher PhA, with graded effects (moderate vs. low: B = 0.420, 95% CI 0.145 to 0.695; *p* = 0.0037; high vs. low: B = 0.554, 95% CI 0.256 to 0.852; *p* < 0.001). These findings indicate that sex and habitual leisure-time activity capture most of the explanatory signal for PhA. Details of the model are presented in Table 4.

Penalized logistic regression (Firth’s method) was then used to reduce small-sample bias and potential separation, with low PhA defined as values below the cohort median, and this confirmed a graded inverse association between free-time physical activity and the odds of low PhA. After adjustment for sex, compared with low free-time activity, moderate activity was associated with significantly lower odds of low PhA (OR = 0.143, 95% CI 0.0139 to 0.809; *p* = 0.0266), and high activity showed an even stronger association (OR = 0.059, 95% CI 0.005 to 0.389; *p* = 0.0023). Male sex was also independently associated with lower odds of low PhA (OR = 0.037, 95% CI 0.000238 to 0.438; *p* = 0.0050). The details are presented in Table 5. Collectively, these findings align with the linear regression results and support habitual leisure-time physical activity as the principal correlate of PhA in this cohort, while also indicating a strong sex-related difference in the probability of low PhA.

Overall, habitual free-time physical activity emerged as the strongest independent correlate of PhA in this cohort.

## 4. Discussion

This study demonstrated that higher levels of free-time physical activity are significantly associated with higher PhA values in adolescents. This relationship remained significant in both linear and penalized logistic regression models. These findings indicate that free-time physical activity is a central correlate of PhA in this cohort, independent of dietary indices or other lifestyle factors. The direction and magnitude of the association are consistent with the interpretation of PhA as a biophysical marker reflecting cellular integrity. Adolescents reporting higher free-time activity may differ in body composition and training-related phenotypes, which could contribute to higher PhA [14]. This warrants direct testing in future work incorporating objective activity metrics and detailed composition measures. Our findings are consistent with previous research in clinical or at-risk populations, where higher PhA has been linked to improved muscular strength, aerobic fitness, and overall health-related fitness [7]. It is worth noting that Ballarin et al. demonstrated in a cohort of children and adolescents with obesity that PhA remained a significant predictor of muscle strength even after adjustment for body composition parameters [7]. This is also consistent with large-scale reference data showing that in children and adolescents, BMI and age are primary determinants of PhA values [22]. These results strengthen the argument that PhA is not merely a reflection of body size or fat-free mass but also a sensitive marker of functional cellular health. This interpretation is consistent with findings in Japanese university students, where PhA was higher in trained athletes compared with nonathletes despite similar fat-free mass, suggesting that habitual physical activity induces qualitative cellular adaptations captured by PhA [4]. While several studies have examined the association between physical activity and PhA, most have focused on clinical populations [23] or adults [11]. This study addresses a notable gap in the literature by focusing on healthy adolescents, for whom normative data and lifestyle factors of PhA remain limited.

By focusing on this group, our study provided baseline data for Central European adolescents, a population for which integrated lifestyle correlates remain comparatively underreported. However, future studies are needed to further investigate this relationship, including frequency, intensity, or type of exercise, as within a single category, different types of activities, such as walking, running, or strength training, could have been included, and these activities differed in intensity and impact on the body. Therefore, our results indicating an association between leisure-time activity and PhA should be interpreted with caution. In a study by Górna S. et al., the actual average number of steps taken by Czech and Polish teenagers during physical education classes was assessed and showed that in both countries, boys were subjected to greater physical exertion during PE lessons than girls. In Central Europe, at least 20 min of intense physical activity is recommended for most types of physical education classes. However, only 25.0% of boys from the Czech Republic and Poland and 18.0% of girls from the Czech Republic and Poland achieved at least 20 min of intense physical activity during physical education classes [24]. In this study, physical activity undertaken at school was not associated with PhA, whereas activity undertaken during leisure time showed such an association. In light of the results presented by Górna et al., the intensity of physical education classes conducted in schools can be questioned. On the other hand, the interpretation of school-related physical activity in our cohort should account for the fact that the school activity variable represents a composite measure rather than physical education exposure alone. In addition to routine movement during the school day, adolescents may engage in extracurricular school-based sports, and the intensity of activity during physical education may vary across students. Therefore, a binary indicator of physical education attendance cannot be assumed to approximate overall activity at school, and that is why we analyzed both these aspects. Consequently, associations involving school-related physical activity should be interpreted as reflecting overall school-time activity patterns, and future studies would benefit from separating compulsory physical education from extracurricular school sport and quantifying intensity and duration using validated instruments or objective monitoring.

What is more, the observed dissociation between school-related activity and PhA, contrasted with the positive association for leisure-time activity, is plausible in light of the Polish school context and the characteristics of adolescent activity constructs. In Polish school education, physical education is compulsory but typically delivered as a limited number of weekly lessons (approximately three hours per week), and the curriculum is designed to promote broad motor competencies and health-oriented physical literacy rather than sustained, high-intensity training; thus, it can impact other health aspects [25]. In parallel, the wider school day remains predominantly classroom-based and thus characterized by prolonged sitting interrupted by brief, low-intensity movement (e.g., walking between classrooms, breaks). Accordingly, although school activity is not strictly sedentary, the overall volume and intensity of school-time movement may be modest. By contrast, leisure-time activity more often represents intentional, higher-intensity, or more frequent engagement (including structured sport) and therefore may provide a stronger and more discriminating exposure signal. This interpretation is consistent with the broader literature indicating that PhA tracks physical fitness and training-related phenotypes in adolescents, including longitudinal evidence demonstrating consistent positive associations between PhA and multiple fitness domains [15,26]. Future studies should therefore consider the objective monitoring (e.g., accelerometry) and separate quantification of physical education intensity, sedentary school time, and extracurricular sport to better disentangle the school versus leisure contributions to PhA. Overall, these findings are consistent with our study and previous studies in which PhA has been identified as a valuable functional marker in adolescents [2].

In contrast, dietary indices derived from frequency-based questionnaires did not show independent associations with PhA after adjustment. This lack of effect should not be interpreted as evidence that nutrition is irrelevant for cellular health. Rather, it may reflect the methodological limitations of self-reported diet indices, which are prone to recall bias and may not capture nutrient density, dietary timing, or energy balance. Moreover, the used questionnaire, while being a validated tool, is dedicated for diet quality assessment and does not quantify total energy intake, portion size, protein intake, or micronutrient adequacy; thus, future studies should also investigate these aspects. In a relatively homogeneous school population, where extreme dietary behaviors are uncommon, the discriminatory ability of such indices may be limited, which leads to limited between-participant variability. Similarly, there are dietary habits among the students or a limited sample size too small to detect subtle associations. Importantly, our study does not rule out the influence of diet on PhA. In addition, global diet quality indices may not adequately represent the pathways most directly linked to PhA (e.g., low-grade inflammation, oxidative stress), especially when the relevant contrast is the degree of food processing rather than healthy vs. unhealthy food frequency patterns. As demonstrated by Detopoulou et al., the key factor may be not the overall composition of the diet but the degree of food processing, as the more processed the food, the lower the PhA, and the less processed, the higher the PhA [27]. This mechanism likely involves ultra-processed foods increasing inflammation and oxidative stress in the body, which potentially worsens cellular health and lowers PhA. What is more, given the sample size and multivariable adjustment, the study may have had limited statistical power and precision to detect small-to-moderate independent effects of dietary indices. Therefore, the absence of statistical significance should be interpreted cautiously and primarily as limited evidence rather than definitive evidence of no association. The lack of an association in our study suggests that future studies should focus on analyzing food processing, not just its overall quality, to better understand the relationship between nutrition and PhA.

While several studies have examined PhA in clinical groups such as adolescents with obesity, malnutrition, or chronic disease, evidence in healthy, community-based adolescent cohorts is scarce [23,28,29]. By focusing on this group, our study addressed a gap in the literature and provided baseline data for Central European adolescents, for whom reference values and lifestyle factors remain poorly described. Unlike prior research focusing on structured exercise programs [15] or total energy expenditure [30], our study specifically investigated habitual free-time activity, thus offering insights into the effects of everyday lifestyle behaviors rather than prescribed training. From a pediatric and public health perspective, these findings are potentially relevant, suggesting that PhA may complement existing indicators in community settings; however, its role as a screening biomarker requires validation in larger, sex-balanced, and preferably longitudinal cohorts. Moreover, the strong and graded relationship with physical activity underscores the importance of promoting active leisure-time behaviors during adolescence, a critical period when lifelong health trajectories are established.

However, several limitations must be acknowledged. The cross-sectional design precludes causal inference. Moreover, while PhA was analyzed as a linear outcome, in our additional analyses with the penalized logistic regression sensitivity analysis, PhA was dichotomized at the cohort median, which reduces information and statistical power, therefore, these results should be interpreted as supportive of robustness rather than as a substitute for the primary continuous-outcome analyses. Lifestyle exposures were self-reported and thus susceptible to recall bias. Pubertal stage was not assessed; although the restricted age range may have mitigated maturational variability, residual confounding by biological maturation cannot be excluded. The chronological age of the participants covered a period in which most adolescents had completed maturation, but significant variability in the rate of biological maturation is still observed in this age group. For this reason, future work should take this aspect into account in order to increase the precision and accuracy of the conclusions. Finally, BIA-derived parameters, including PhA, depend on measurement standardization, though all assessments followed a single protocol administered by trained staff.

Moreover, in the present study, the relatively modest sample size and the pronounced sex imbalance should be explicitly acknowledged as key constraints on inference, particularly for the interpretation of sex-specific effects. Although male sex emerged as a strong predictor of higher PhA, this estimate was derived from a limited number of male participants. Consequently, the observed sex coefficient should be interpreted as exploratory rather than confirmatory, and it should not be used to imply population-level sex differences in Central European adolescents without replication in a sex-balanced cohort. Importantly, sex differences in adolescent PhA are biologically plausible and have been reported in prior adolescent studies, but available evidence indicates that these differences are tightly intertwined with body size and lean mass, which may confound or amplify apparent sex effects when one sex is markedly underrepresented [15,26]. In this context, recent longitudinal evidence focused specifically on male adolescents provides a useful comparator, as in Brazilian male adolescents, de Moraes et al. reported longitudinal associations between physical fitness components and PhA, supporting the concept that PhA reflects functional somatic status and cellular health in adolescence [26]. Taken together, our findings are consistent with the broader direction of the literature, yet they primarily provide hypothesis-generating baseline data, and future studies should prioritize larger samples with balanced sex distribution and, ideally, longitudinal follow-up incorporating maturation and fitness markers to disentangle behavioral correlates from developmental and body-composition drivers of PhA.

Despite these limitations, the study adds to the limited pediatric literature by integrating validated diet and activity measures with BIA-derived PhA in healthy adolescents. Prospective studies using objective activity monitoring and more detailed dietary assessment are warranted to clarify temporal relationships and to test whether changes in free-time activity translate into measurable PhA differences over time.

## 5. Conclusions

In healthy adolescents, free-time physical activity is the main lifestyle correlate of PhA, whereas self-reported diet quality indices show no independent association. Promoting active free-time behaviors may be associated with more favorable PhA profiles reflecting cellular health. However, longitudinal studies are required to determine whether increasing activity produces measurable changes in PhA over time.

## Figures and Tables

**Table 1:** Characteristics of the study group.

Variable (Unit)	Mean ± SD/Median (IQR)
Age (years)	16 (16.0–17.0)
BMI (kg/m^2^)	20.63 (18.81–22.68)
Waist circumference (cm)	69 (63.25–75)
Phase angle (°)	5.16 (4.88–5.46)
Fat mass (%)	13.15 (11–17.6)
Muscle mass (kg)	0.45 (0.39–0.49)
Fat-free mass (kg)	43.35 (39.57–46.82)
Total body water (kg)	29.45 (27.68–31.75)
Body cell mass (kg)	0.53 (0.51–0.56)
pHDI	18.85 (11.3–24.4)
nHDI	16.18 (11.82–20.38)
DQI	3.08 ± 12.67
Eggs (servings/day)	0.14 (0.06–0.50)
Legumes (servings/day)	0.06 (0–0.06)
White meat (servings/day)	0.50 (0.14–0.50)
Red meat (servings/day)	0.06 (0.01–0.14)
Fermented milk products (serv/day)	0.14 (0.06–0.50)
Wholegrain bread (servings/day)	0.50 (0.06–0.50)
Plain pasta (servings/day)	0.50 (0.14–0.50)
Wholegrain pasta (servings/day)	0.14 (0.06–0.50)
Sweets (servings/day)	0.50 (0.50–1.00)
Fast food (servings/day)	0.06 (0.06–0.14)
Sex	Female: 48 (85.7%), Male: 8 (14.3%)
Physical activity at free time	Low: 14 (30.4%), Moderate: 18 (39.1%), High: 14 (30.4%)
Physical education class attendance	No: 8 (17.4%), Yes: 38 (82.6%)
Sleep weekdays	<6 h: 21 (46.7%), 6–9 h: 23 (51.1%), >9 h: 1 (2.2%)
Sleep weekends	<6 h: 1 (2.2%), 6–9 h: 24 (52.2%), >9 h: 21 (45.7%)

pHDI—pro-healthy diet index, nHDI—non-healthy diet index, DQI—diet quality index.

**Table 2:** Univariate regression results between PhA and investigated parameters.

Variable (Unit)	Estimate	*p*-Value
Male sex	+1.021	<0.001
Physical activity level at free time	+0.380	<0.001
Physical activity level at school	−0.103	0.513
Physical education class attendance	+0.198	0.402
Age (years)	+0.036	0.660
Sleep time on weekdays	+0.141	0.406
Sleep time on weekends	−0.028	0.867
BMI (kg/m^2^)	+0.067	0.0070
nHDI (points)	+0.0047	0.724
pHDI (points)	+0.0116	0.133
DQI (points)	+0.0084	0.238
Plain pasta consumption (servings/day)	+1.118	0.0019
Canned meat consumption (servings/day)	+3.192	0.0057
Wholegrain pasta consumption (servings/day)	+0.878	0.0062
Legumes consumption (servings/day)	+1.703	0.0195
Eggs consumption (servings/day)	+0.550	0.0267
White meat consumption (servings/day)	+0.560	0.0347
Red meat consumption (servings/day)	+0.827	0.0383
Fermented milk products consumption (servings/day)	+0.551	0.0394
Wholegrain bread consumption (servings/day)	+0.385	0.0495
Hot drinks consumption (servings/day)	−0.159	0.181
Nuts consumption (servings/day)	+0.898	0.236
Water consumption (servings/day)	+0.164	0.293
Fried meals consumption (servings/day)	+0.213	0.312
Potatoes consumption (servings/day)	+0.370	0.354
White bread consumption (servings/day)	−0.134	0.379
Sweets consumption (servings/day)	−0.127	0.407
Butter consumption (servings/day)	−0.097	0.496
Energy drinks consumption (servings/day)	+0.242	0.507
Milk consumption (servings/day)	−0.099	0.545
Vegetables consumption (servings/day)	+0.067	0.579
Cheese consumption (servings/day)	+0.103	0.619
Fruit juices consumption (servings/day)	−0.101	0.619
Alcohol consumption (servings/day)	+1.139	0.644
Canned vegetables consumption (servings/day)	+0.083	0.769
Margarine consumption (servings/day)	+0.106	0.795
Vegetable juices consumption (servings/day)	+0.087	0.800
Fruits consumption (servings/day)	+0.030	0.817
Ready-made soups consumption (servings/day)	−0.104	0.825
Fast food consumption (servings/day)	−0.141	0.846
Processed meat consumption (servings/day)	−0.029	0.854
Sweetened drinks consumption (servings/day)	+0.049	0.885
Lard consumption (servings/day)	+0.050	0.922
Quark consumption (servings/day)	−0.009	0.975
Fish consumption (servings/day)	+0.010	0.991

pHDI—pro-healthy diet index, nHDI—non-healthy diet index, DQI—diet quality index, BMI—body mass index.

**Table 3:** Detailed multivariable regression model predicting PhA.

Predictor	Estimate	95% CI	*p*-Value
Male sex	+0.90	[3.498; 5.307]	<0.001
BMI (kg/m^2^)	+0.01	[0.464; 1.33]	0.601
Free-time physical activity (moderate vs. low)	+0.31	[−0.033; 0.057]	0.066
Free-time physical activity (high vs. low)	+0.53	[−0.022; 0.635]	0.0076
Plain pasta consumption (servings/day)	+0.61	[0.151; 0.909]	0.090
Wholegrain pasta consumption (servings/day)	−0.03	[−0.102; 1.325]	0.922
Legumes consumption (servings/day)	−0.05	[−0.66; 0.599]	0.936
Eggs consumption (servings/day)	−0.33	[−1.383; 1.276]	0.328
White meat consumption (servings/day)	+0.04	[−0.994; 0.342]	0.881
Red meat consumption	+0.11	[−0.463; 0.537]	0.755
Fermented milk products consumption (servings/day)	+0.06	[−0.621; 0.848]	0.846
Wholegrain bread consumption (servings/day)	−0.00	[−0.571; 0.692]	0.979

BMI—body mass index.

**Table 4:** Reduced multivariable linear regression model predicting PhA.

Predictor	Estimate	95% CI	*p*-Value
Male sex	+0.96	[4.542; 4.955]	<0.001
PA (moderate vs. low)	+0.42	[0.655; 1.268]	0.0037
PA (high vs. low)	+0.55	[0.145; 0.695]	0.0005

**Table 5:** Penalized logistic regression model predicting low PhA.

Predictor	OR	95% CI	*p*-Value
Male sex	0.037	[0.000238; 0.438]	0.0050
PA (moderate vs. low)	0.143	[0.0139; 0.809]	0.0266
PA (high vs. low)	0.059	[0.005; 0.389]	0.0023

PA—physical activity.

## Data Availability

The dataset from this study is available from the authors upon reasonable request.
